# The TerC family metal chaperone MeeY enables surfactin export in *Bacillus subtilis*

**DOI:** 10.1128/jb.00088-25

**Published:** 2025-04-16

**Authors:** Bixi He, Ankita J. Sachla, Sadie B. Ruesewald, Daniel B. Kearns, John D. Helmann

**Affiliations:** 1 Department of Microbiology, Cornell University 5922, Ithaca, New York, USA; 2 Department of Biology, Indiana University 1772, Bloomington, Indiana, USA; University of Massachusetts Chan Medical School, Worcester, Massachusetts, USA

**Keywords:** surfactin, swarming, *Bacillus subtilis*, TerC family, manganese

## Abstract

TerC family proteins are widely conserved integral membrane proteins with functions related to metal transport. In *Bacillus subtilis*, the TerC proteins MeeF and MeeY play overlapping roles in the metalation of manganese-requiring membrane and extracellular enzymes. TerC proteins interact with the secretion translocon SecYEG and metalate proteins either during or after protein translocation. Here, we demonstrate that swarming motility is dependent on MeeY. This swarming defect can be complemented extracellularly and is correlated with a loss of surfactin. Surfactin export is mediated by SwrC, an RND family efflux pump previously shown to interact with MeeY in co-immunoprecipitation studies. The amendment of the growth medium with manganese has long been known to enhance surfactin production. We suggest a model in which surfactin export is enhanced by the MeeY-dependent metalation of the surfactin lipopeptide during export.

**IMPORTANCE:**

*Bacillus subtilis* produces surfactin, a powerful detergent-like compound that functions in intercellular communication, surface motility, and as a broad-spectrum antimicrobial agent. Production of surfactin, a cyclic lipopeptide, depends on a non-ribosomal peptide synthase followed by export by SwrC, a member of the resistance-nodulation-cell division (RND) family of export proteins. Here, we demonstrate that surfactin production additionally requires MeeY, a TerC family membrane protein that exports manganese ions to support the function of secreted and membrane metalloenzymes. We propose that MeeY interacts with SwrC to facilitate metal binding to the surfactin lipopeptide during export from the cell. These results may explain the long-appreciated role that divalent metal ions play in surfactin production during industrial fermentation.

## INTRODUCTION

TerC family proteins are conserved membrane proteins with seven transmembrane segments and are implicated in metal transport (1, 2). TerC proteins were originally identified in operons that confer resistance to the toxic metalloid tellurite (3, 4). However, the role of TerC proteins in resistance is unclear. The presence of TerC proteins in diverse organisms, most of which are unlikely to encounter toxic levels of tellurite, suggests a broader function. TerC proteins are members of the LysE superfamily (5), which includes proteins implicated in the transport of calcium (Ca^2+^) or manganese (Mn^2+^) (2, 5). Notable representatives include the *E. coli* Alx protein (2, 6), the *Arabidopsis* TerC (AtTerC) protein important for the synthesis of photosynthetic complexes (7), and the human TMEM165 protein required for efficient protein glycosylation in the Golgi complex (8).

*B. subtilis* encodes two TerC proteins (MeeF and MeeY) with overlapping functions in Mn export (9, 10). A third TerC paralog, YjbE, is expressed during sporulation and has yet to be characterized (11). In previous work, we described the role of two cation diffusion facilitator (CDF) family efflux proteins, MneP and MneS, in protecting cells against Mn^2+^ toxicity (12). Mutant strains lacking both proteins (*mneP mneS*) are highly sensitive to Mn^2+^, but this sensitivity is reduced if the expression of the TerC protein MeeF (formerly YceF) is elevated (9). Furthermore, both MeeF and MeeY (formerly YkoY) function together in reducing intracellular Mn^2+^ levels (9). These studies support the idea that the MeeY and MeeF TerC proteins export Mn^2+^ from cells, but since this function is not needed for Mn^2+^ resistance in cells that have the MneP and/or MneS proteins, their physiological function was not immediately apparent.

Subsequent work revealed that the MeeF and MeeY proteins function to deliver Mn^2+^ across the cell membrane to assist in the maturation of Mn-requiring membrane and secreted proteins (10). In the *meeF meeY* double mutant (*FY* mutant), protein secretion was dramatically decreased, an effect proposed to result from jamming of the SecYEG secretion translocon by proteins that fail to get properly metalated during secretion (10). To date, the major phenotypes linked to defects in TerC function in *B. subtilis* include poor growth on lysogeny broth (LB), due largely to decreased production of secreted feeding proteases, and reduced activity of lipoteichoic acid synthase (LtaS), an important cell envelope synthesis enzyme with an extracellular active site containing a catalytic Mn ion (10). This role for TerC proteins is likely conserved in related bacteria, as recently suggested for a MeeY ortholog (MntY) from *Staphylococcus aureus*, which is also required for LtaS function (13).

Here, we have explored the impact of TerC proteins using the undomesticated *B. subtilis* NCIB3610 strain (3610). Unlike the commonly used laboratory strains based on *B. subtilis* 168, strain 3610 displays more complex developmental phenotypes, including swarming motility. However, swarming was absent in the 3610 *meeY* mutant due to a defect in the production of surfactin, a surface-active lipopeptide required for swarming motility. Since MeeY interacts with the SwrC surfactin export protein (10), we suggest that surfactin release from the cell is dependent on the TerC-dependent binding of Mn to the surfactin lipopeptide.

## RESULTS

### The MeeY protein is essential for swarming motility

To further explore the roles of *B. subtilis* TerC paralogs, we generated single, double, and triple mutant strains in the *meeF*, *meeY*, and *yjbE* genes in the undomesticated NCIB 3610 strain (3610), which displays both swimming and swarming motility (14). Although *B. subtilis* 168 strains are motile in liquid, they fail to swarm on plates with agar concentrations of 0.7% (15). This defect is a consequence of two loss-of-function frameshift mutations in the 168 lineage: *sfp^0^* inactivates a phosphopantetheinyl transferase required for surfactin synthesis, and *swrA* inactivates an essential co-activator of the major *fla/che* operon required for the production of swarming proficient cells (16, 17). The CU1065 strain that we routinely use for studies of metal ion physiology is a 168 derivative (18).

**Fig 1:**
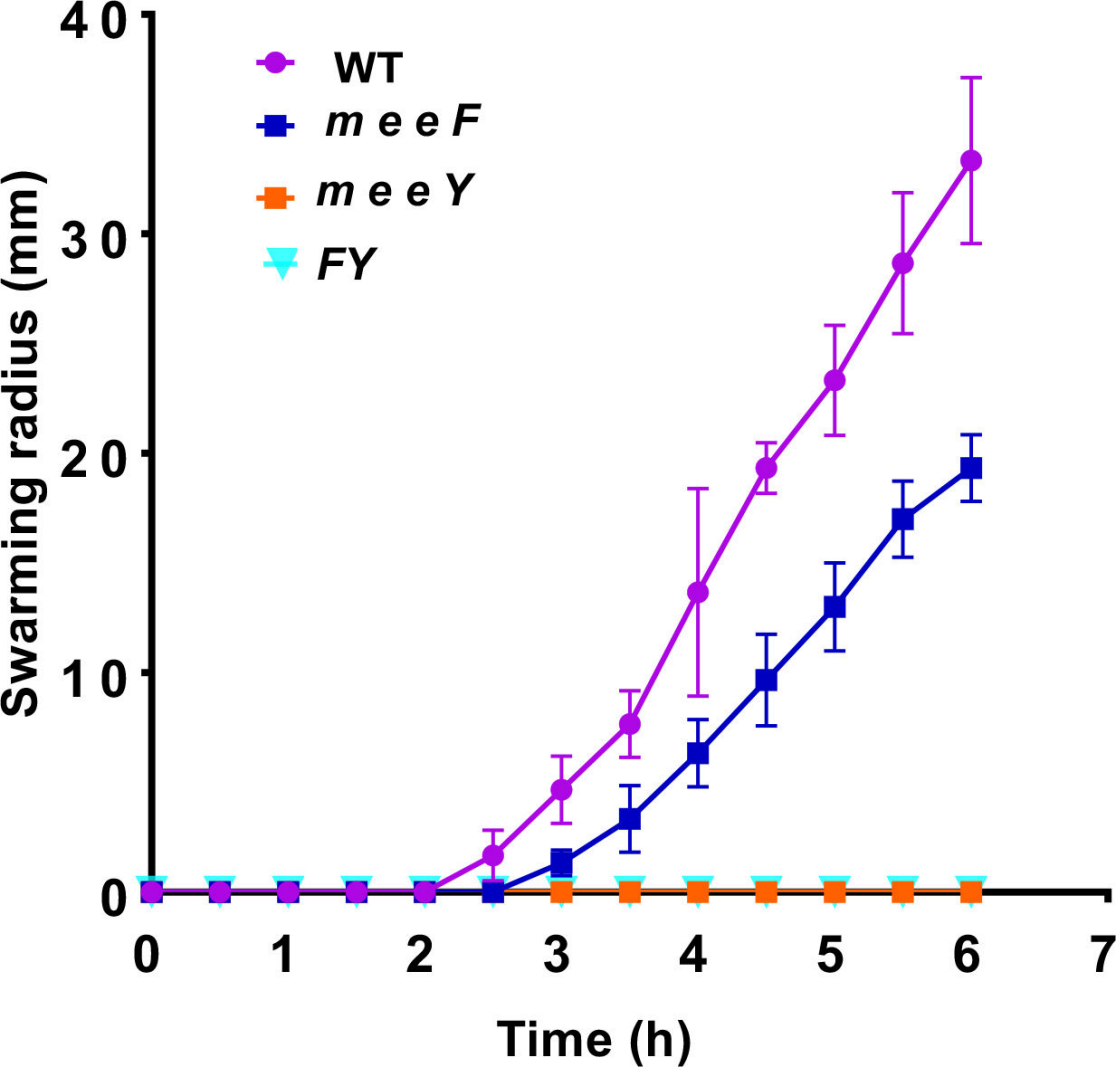
The *meeY* mutant is defective in swarming. Swarm expansions of 3610 and mutants were quantitated on LB agar plates. Swarming diameter of WT, *meeF*, *meeY*, and *meeF meeY* (*FY*) mutants on 0.7% agar LB plates at 37°C. Results shown are from three replicate plates spotted from the same inoculum (mean ± SD at each time point; *n* = 3) and are representative of results repeated with >3 independent biological replicates.

We spot-inoculated bacteria on Petri plates (0.7% agar) and measured the radius of the swarm as a function of time. Swarming motility was lost in mutant strains lacking *meeY* either alone or in the FY double mutant (Fig. 1; Fig. S1). By contrast, the *meeF* single mutant had a modest, but reproducible, reduction in swarming diameter as a function of time. The *meeY* swarming defect was at least partially complemented by the ectopic expression of *meeY* under the control of a xylose-inducible promoter (Fig. S2). The incomplete rescue may reflect the fact that *meeY* is normally under multiple levels of control, including induction from an Mn^2+^-sensing riboswitch (19, 20).

To determine whether *meeF* and/or *meeY* are also important for swimming motility, we first moved these mutations into a background (*srfAC::Tn10* Δ*epsH*) that eliminates swarming motility due to loss of surfactin biosynthesis (21) and sliding motility due to lack of extracellular polysaccharide (22, 23). In this background, both the *meeY* and *meeF* single mutants displayed normal swimming motility. However, the *FY* double mutant had greatly reduced swimming motility (Fig. S3). Since the *FY* mutant is pleiotropic and has impaired growth on LB medium (10), we here focus on the swarming defect of the *meeY* single mutant.

### The *meeY* swarming defect is not due to a loss of flagellar synthesis

**Fig 2:**
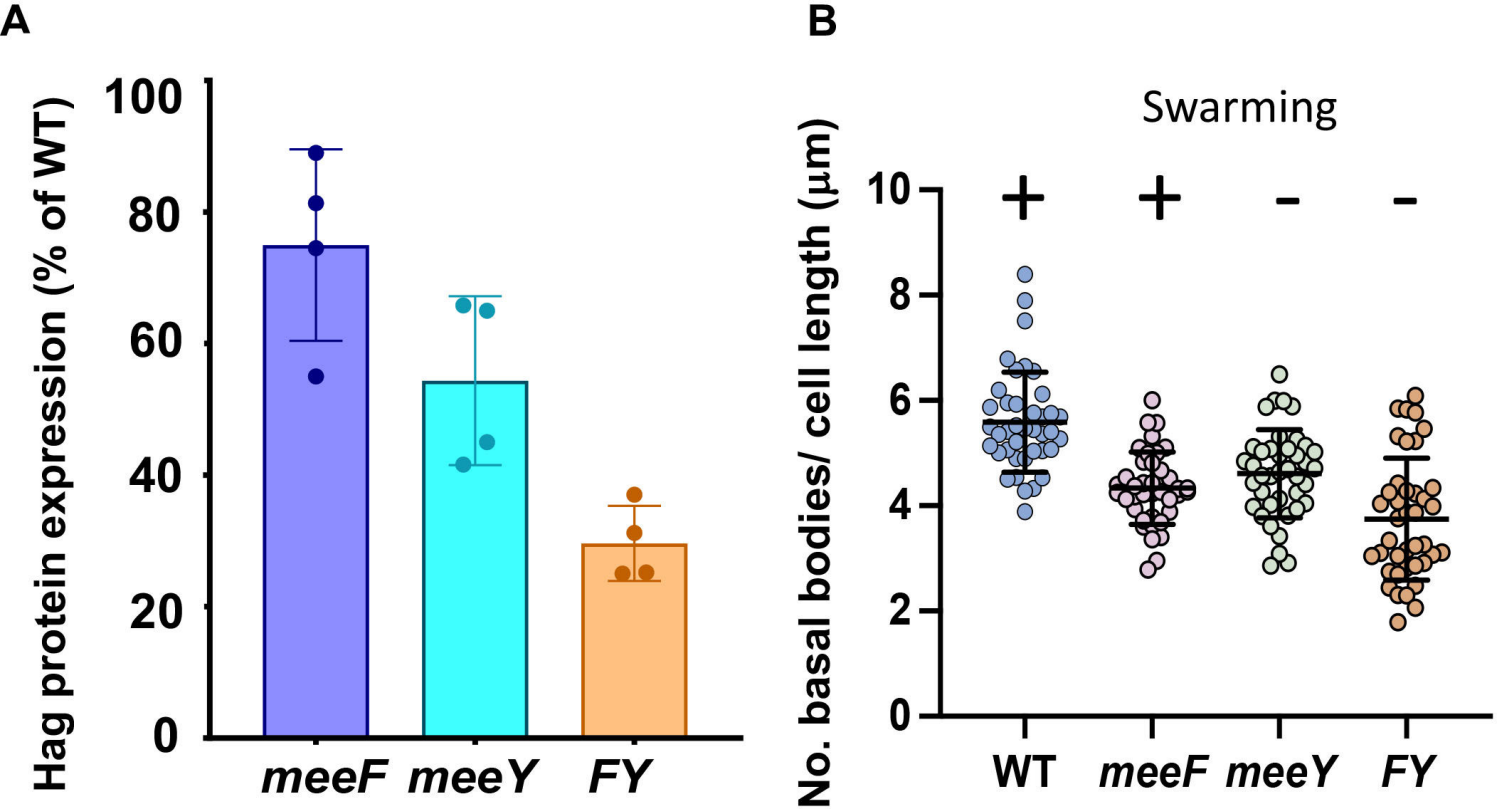
Defects in flagella assembly are not well correlated with swarming proficiency. (**A**) Flagellin (Hag) protein levels were quantified by Coomassie-stained SDS-PAGE, and the relative intensities of the Hag band were compared to WT (*n* = 3) (see also Fig. S4). The reduction seen in the *meeY* mutant relative to WT is significant (based on an ANOVA multiple comparisons and Tukey’s test, *P*-value < 0.001). (**B**) The number of basal bodies normalized to cell length (µm) (mean ± SD; *n* = 40; see also Table S1).

Compared to swimming bacteria, swarming cells have a greater number of flagella per cell (14). Since *FY* mutants were previously shown to be defective in protein secretion through the general secretory pathway (10), we hypothesized that the *meeY* strain might have a reduced capacity for the synthesis of flagella. While much of the flagellar structure is assembled from substrates translocated through the hollow rod and filament by a specialized type III protein export apparatus, the initial assembly of the basal body relies on transmembrane components secreted by the general secretory pathway (24). To monitor flagellar synthesis, we measured flagellin (Hag protein) levels in the 3610 WT and mutant derivatives by SDS-PAGE. Quantitation of the Coomassie-stained protein band revealed a modest, albeit significant, decrease (<2-fold) in the *meeF* and *meeY* single mutants, and a more severe decrease in the *FY* mutant (Fig. 2A; Fig. S4). As expected, flagellin was undetectable in the *hag::erm* mutant (Fig. S4). Since both *meeF* and *meeY* mutants had modest decreases in flagellin levels, yet only *meeY* affected swarming, we conclude that decreased flagellar filament levels are unlikely to be solely responsible for the *meeY* swarming defect.

The reduced level of flagellin in the mutant strains could result from either fewer flagella per cell or a change in the average length of flagellar filaments. Therefore, we used three-dimensional structured illumination microscopy (3D-SIM) to quantify the number of basal bodies in individual cells. For this purpose, we used a 3610 derivative (∆*fliM amyE::P_flache_-fliM-GFP*) with a GFP-tag appended to the FliM protein in the C-ring of the basal body (25) in WT and isogenic *meeF*, *meeY,* and *FY* mutants. Quantitation of basal bodies per cell revealed no significant difference between the 3610 strain and the *meeF* (swarming competent) and *meeY* (swarming deficient) derivatives (Table S1). Further analyses revealed that the cells lacking one or more TerC paralogs had somewhat longer cell lengths compared to WT (Table S1), and a modestly reduced number of basal bodies per cell length, which results in a modest reduction of basal bodies per μm of cell length (Fig. 2B). There was no apparent difference between the *meeY* and *meeF* single mutants (Fig. 2B), even though *meeF* swarms well and *meeY* does not (Fig. 1). We conclude that the modest reduction in the density of basal bodies per unit cell length does not correlate with swarming proficiency (Fig. 2B).

### The *meeY* swarming defect can be complemented extracellularly

**Fig 3:**
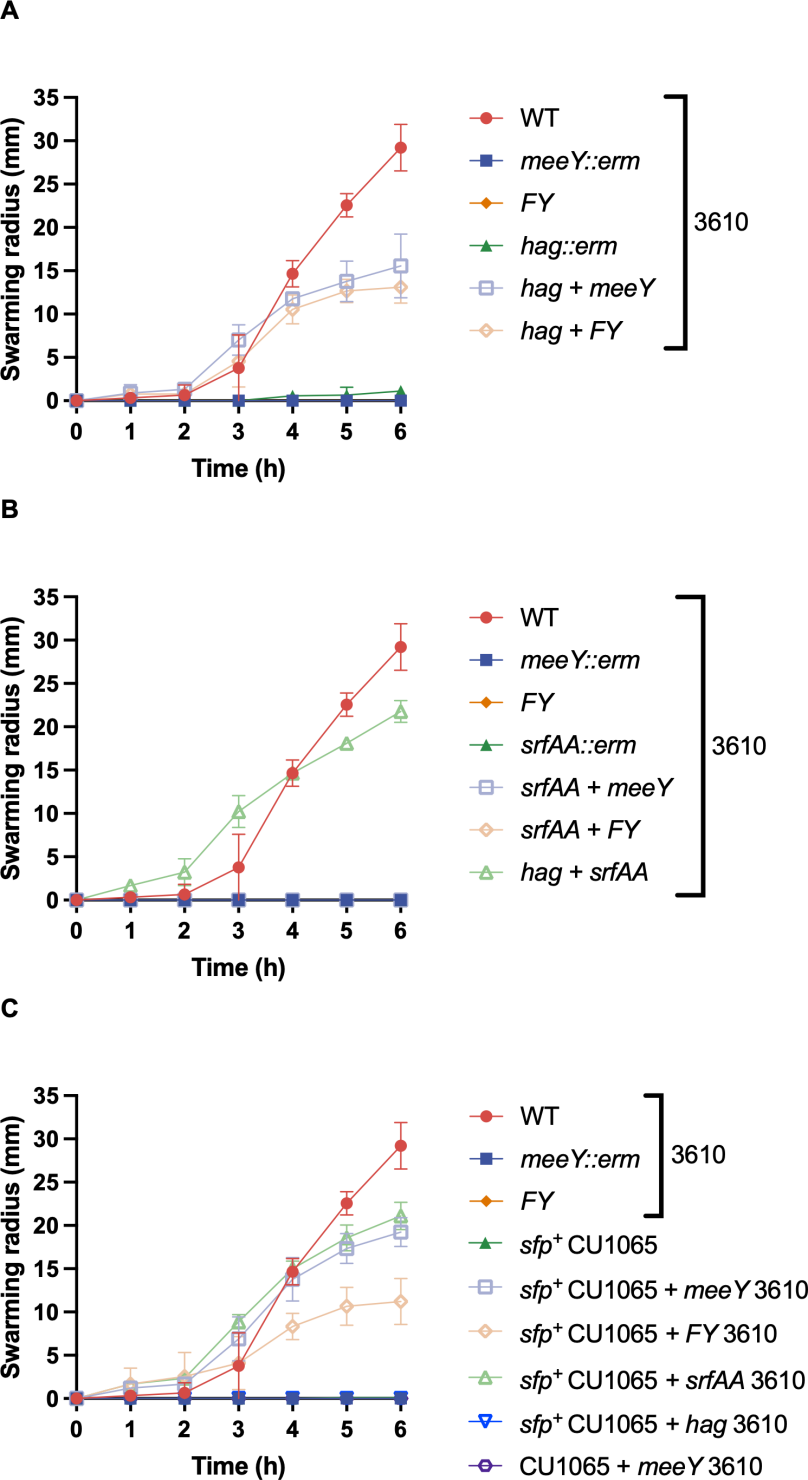
The swarming defect of the *meeY* mutant can be complemented extracellularly by surfactin-producing strains. Swarm expansions of mixed cultures (1:1) were quantitated after spot inoculation of 10 µL cell suspension on 0.7% LB agar plates. (**A**) A *hag* mutant can restore the defective swarming motility of *meeY* and *meeF meeY* (*FY*) mutants. (**B**) 3610 *srfAA* strains cannot complement *meeY* and *FY* mutants. (**C**) The surfactin-producing CU1065 strain *sfp^+^* + restores swarming motility to *meeY* and *FY*. Data are from three independent experiments and presented as mean ± SD.

Some mutations that lead to swarming defects can be complemented extracellularly (26). These include mutations that impair the synthesis of surfactin, a lipopeptide with detergent-like properties that are essential for surface motility. When the swarming-deficient *meeY* or *FY* mutants were co-inoculated with a swarming-defective 3610 *hag* null mutant, swarming was restored (Fig. 3A). The flagellated, but swarming-defective *meeY* and *FY* mutant strains swarmed at a near-normal rate for ~4 hours, and then the swarm slowed. This is consistent with a limited ability of surfactin to diffuse from the non-motile *hag* mutant cells at the site of inoculation. Consistent with this interpretation, extracellular complementation was not observed with the *meeY* or *FY* mutant strains when they were mixed with a 3610 *srfAA* derivative (Fig. 3B), supporting the idea that *meeY* mutant strains do not produce surfactin. Furthermore, extracellular complementation of *meeY* was not observed with the CU1065 strain which fails to make surfactin. Correction of the *sfp^0^* mutation in this strain is known to restore surfactin synthesis but not motility since this strain is also defective in *swrA*. This corrected strain (CU1065 *sfp*^+^) was now able to complement the swarming defect of the *meeY* and *FY* strains (Fig. 3C).

**Fig 4:**
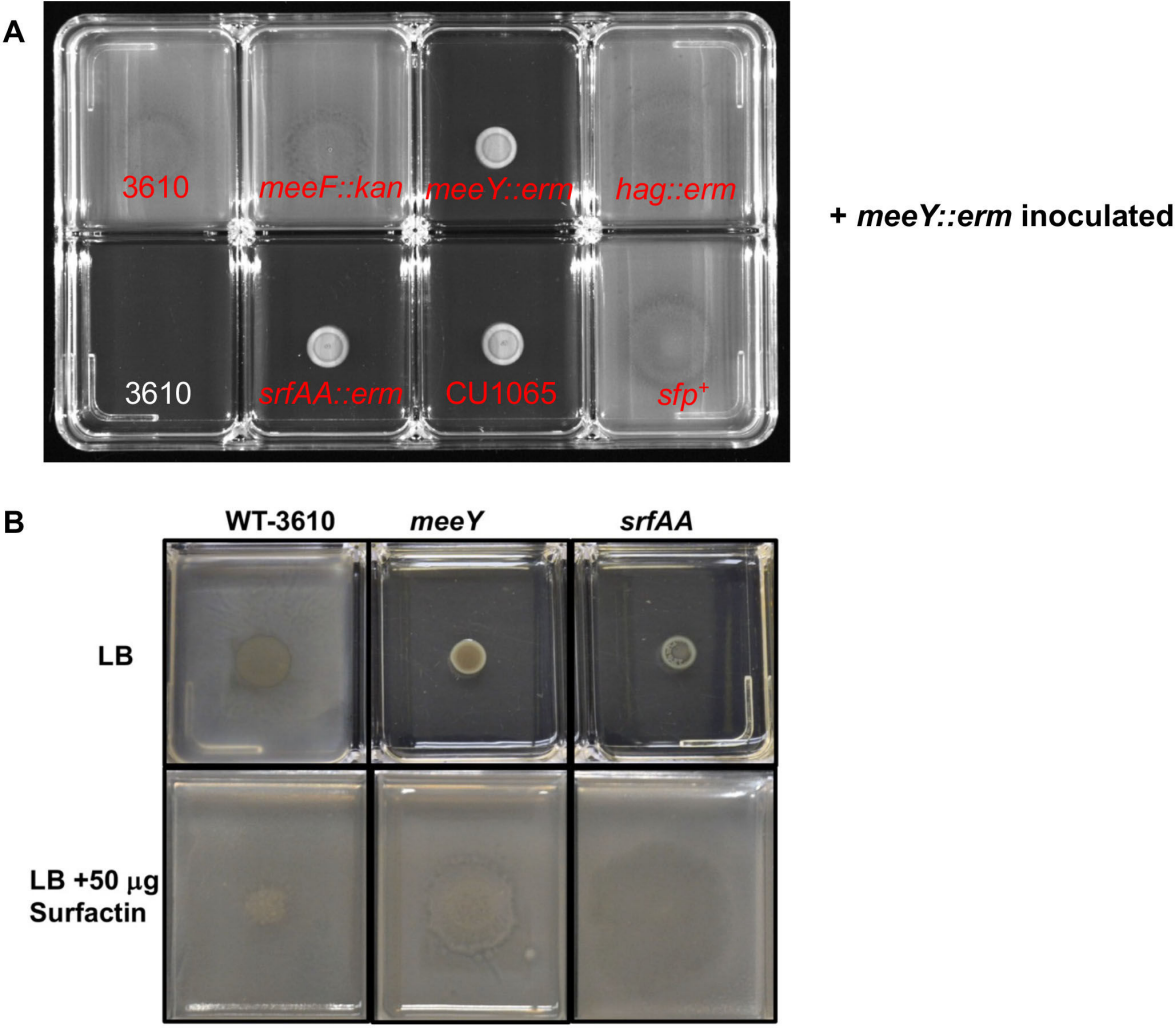
Extracellular complementation is observed with cell-free supernatants from surfactin-producing strains. (**A**) Supernatants were collected from different cultures (grown in LB to OD_600_ = 1), filter-sterilized, and tested for their ability to restore swarming to spot-inoculated *meeY* cells (5 µL). The source of the supernatant fraction is indicated on the plates in red. The supernatants were from 3610 (upper left) or derivative strains, except for CU1065 and a CU1065 *sfp*^+^ derivative (bottom row). The lower left panel is a sterility control with 3610 supernatant only with LB agar and no cells added. Similar results were seen with the *meeF meeY* (*FY*) double mutant (see Fig. S5). (**B**) 5 µL of WT, *meeY* or *srfAA* cell resuspensions were inoculated onto LB with or without 50 mg of surfactin (Sigma chemical, S3523). Both panels are 8-well plates (Nunc rectangular #176600; each well is 26 × 33 mm) incubated overnight at 37°C. All images are representative results from three independent biological replicates.

To confirm that the extracellular complementation was due to a secreted product (surfactin) and did not require cell-to-cell contact, we tested the ability of filter-sterilized cell supernatants to restore motility (Fig. 4A). Complementation was observed with supernatant fractions from all strains expected to produce surfactin (3610, 3610 *meeF*, 3610 *hag*, and CU1065 *sfp*^+^) but not with supernatant fractions from those that do not produce surfactin (3610 *srfAA*, CU1065 *sfp^0^*). These results suggest that the failure of *meeY* to swarm is due specifically to a loss of surfactin production. We further demonstrate that the addition of purified surfactin can restore swarming motility to the *meeY* strain (Fig. 4B).

## DISCUSSION

Because of its commercial potential, the parameters affecting surfactin production have been extensively studied over decades (27). However, surfactin remains a niche product. Compared to the 17 million metric tons of surfactants produced annually (~$50 billion market value), microbially produced lipopeptides together with other green “biosurfactants” account for <5% of the market share (28). Surfactin is useful in cosmetic, personal care, and pharmaceutical applications, with production costs requiring a minimum selling price of ~$30 /kg (28). Early studies revealed a key role of metal ions in general, and Mn in particular, in supporting optimal surfactin production (29). Despite the routine amendment of culture medium with Mn, the mechanism by which Mn enhances the yield of surfactin has never been revealed. Our results imply that MeeY, a TerC family Mn exporter, is required for efficient production of surfactin.

TerC family proteins are conserved membrane proteins (COG0861) found in all three domains of life and implicated in a wide variety of functions. Bacterial TerC homologs were first identified in operons linked to tellurite resistance often found on plasmids (30). Chromosomally encoded TerC paralogs in *E. coli* (Alx) and *B. subtilis* (MeeY, formerly YkoY) are both regulated by a *yybP/ykoY* family riboswitch, with expression induced in response to elevated intracellular Mn (19, 20). The *E. coli* Alx protein also supports Mn export and is activated by high pH, which mobilizes intracellular Mn pools (6). The *B. subtilis* MeeY protein and its paralog MeeF both function in Mn export in support of the metalation of extracytoplasmic enzymes (9, 10). In *B. subtilis* 168, the *meeF meeY* double mutant, but not the single mutants, displays a significant growth defect, has reduced protein secretion due to jamming of the SecYEG translocon, and is defective in metalation of lipoteichoic synthases (10). A similar role in LtaS metalation has recently been proposed for MntY, a MeeY ortholog from *S. aureus* (13).

Here, we report that MeeY is required for swarming motility in *B. subtilis* 3610. Mutants lacking MeeY are swarming defective due to a lack of exported surfactin, a lipopeptide with potent surfactant activity. Surfactin synthesis is mediated by a large, non-ribosomal protein synthesis (NRPS) enzyme complex encoded by the *srfAA-AB-AC-AD* operon, which is itself under complex transcriptional regulation (31). Once surfactin biosynthesis is complete, the mature lipopeptide is exported from the cell by the SwrC (YerP) transport protein (32), and possibly by other proteins such as YfiS, a member of the major facilitator superfamily (33). We can now extend this model to include a critical role for the MeeY protein in the production or export of surfactin.

**Fig 5:**
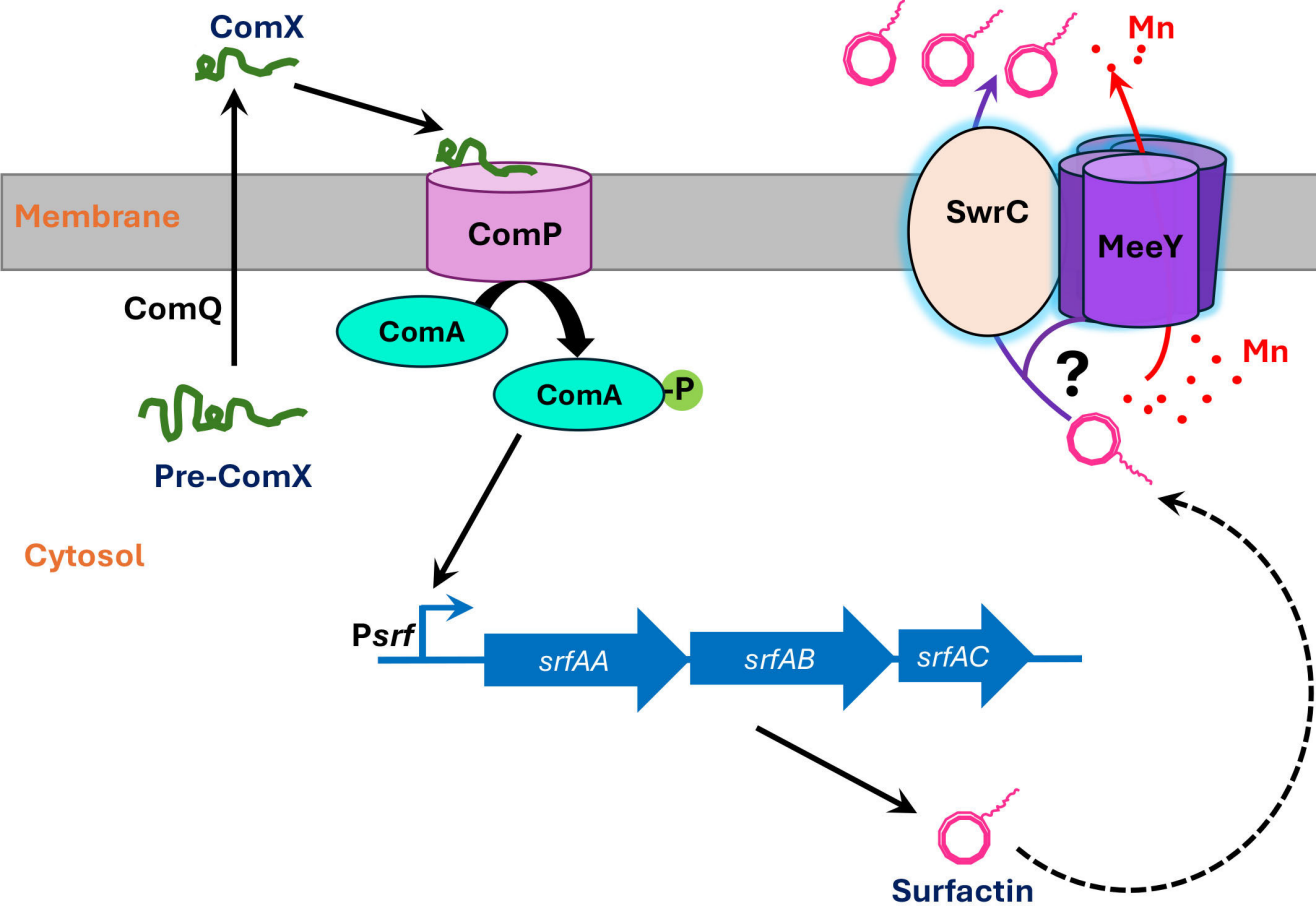
The MeeY membrane protein is required for the efficient secretion of the surfactin lipopeptide. Expression of the surfactin biosynthesis operon is controlled by the ComPA two-component regulatory system that responds to extracellular ComX peptide. The surfactin operon encodes a large, non-ribosomal protein synthase (NRPS) that requires modification by the Sfp phosphopantetheinyl transferase. Surfactin secretion depends on the SwrC membrane protein, which likely functions as an exporter. We postulate that MeeY metalates the surfactin lipopeptide to facilitate its release from SwrC, and in the absence of MeeY, this final step in export is defective, leading to greatly reduced surfactin activity in the supernatant fraction.

One model consistent with our data is that surfactin secretion could be coupled to metal-loading by MeeY (Fig. 5). Surfactin has long been known to form complexes with divalent metal ions, and in early purification protocols, Ca^2+^ was added to facilitate crystallization (34). The binding affinity of surfactin for Ca^2+^ is relatively weak, with measured *K*_d_ values in the 5–10 μM range (35, 36). Although the affinity for Mn^2+^ has not been reported, Mn^2+^ often binds 10–100× tighter than Ca^2+^ to simple organic ligands (37). We hypothesize that MeeY may load Mn into surfactin as it exits the cell, and the resulting conformation change may be needed to efficiently release the lipopeptide from cells. Once released from the cell, the surfactin may or may not retain a bound cation depending on the cation concentration in the medium. A role for MeeY in the metalation of surfactin is analogous to the proposed role of the MeeY and MeeF proteins in the co-translocational loading of metal ions into ribosomally synthesized secretory proteins (10).

The details of surfactin export are still not well resolved. SwrC, a member of the resistance-nodulation-cell division (RND) family of proton-coupled efflux proteins, has been most strongly implicated in export during swarming (21). In addition, surfactin export is inhibited by chemical inhibitors of the proton gradient, and overproduction of SwrC significantly increases surfactin production (32). The co-immunoprecipitation (Co-IP) of SwrC with MeeY-FLAG protein (10) is consistent with a physical interaction between the two proteins. It is also intriguing that the surfactin synthetase proteins were recovered in the Co-IP experiment, suggesting that the synthetase may also interact with the secretion complex. One puzzle yet to be resolved is that SwrC is also recovered in the Co-IP with MeeF-FLAG protein, even though MeeF protein alone cannot support surfactin production. One possible explanation is that the TerC proteins may function as either homo- or heterooligomers, and complexes with both MeeY and MeeF may support surfactin export, thereby accounting for the recovery of SwrC in Co-IP with both TerC paralogs.

While our evidence to date has linked MeeY and MeeF proteins to Mn export, other TerC-related proteins transport Ca^2+^ instead of or in addition to Mn^2+^ (2). These include members of uncharacterized protein family 0016 (UPF0016) such as human Golgi protein TMEM165 (38–40). The nature of the metal exported by MeeY that is required for surfactin production is not yet clear, but given the evidence that MeeY and MeeF export Mn^2+^ (10, 41), we favor the idea that Mn^2+^ is the relevant ion. Although several biophysical studies have characterized the effects of divalent cations on surfactin structure and activity (36, 42, 43), these studies have generally focused on Ca^2+^ rather than Mn^2+^. However, it is likely that surfactin can bind other divalent cations. For example, treatment of *B. subtilis* with purified surfactin induces genes associated with zinc depletion (44). Further studies of the metal selectivity of TerC family metal exporters and characterization of the ion coordination by natively secreted surfactin will be helpful to resolve this point.

## MATERIALS AND METHODS

### Bacterial strains and growth conditions

All strains used in this study are listed in Table S2. Mutant strains in the 168 background were obtained from the *Bacillus* Genetic Stock Center (BGSC) as erythromycin or kanamycin-marked gene disruptants from the BKE or BKK collection (45). Mutations were transformed into CU1065 (a 168 derivative; (18)) and markerless in-frame mutants were generated by transformation with plasmid pDR244 to remove the erythromycin or kanamycin cassette (45). The *sfp*^+^ derivative of CU1065 was generated using congression (46). Mutants in the NCIB 3610 background were constructed using SPP1-mediated generalized phage transduction. Gene deletions were confirmed by PCR analysis using flanking or internal primers (Table S3).

### Growth conditions

Bacteria were grown in liquid or on solid lysogeny broth (LB) (RPI) at 37°C unless otherwise stated. LB medium contains 10 g tryptone, 5 g yeast extract, and 5 g NaCl per liter. Antibiotics used for selecting *B. subtilis* strains include the following: spectinomycin 100 µg/mL, macrolide-lincosamide-streptogramin B (MLS = 1 µg/mL erythromycin +25 µg/mL lincomycin), kanamycin 15 µg/mL. MC medium for transformation includes: 100 mM K_2_HPO_4_, 100 mM KH_2_PO_4_, 3 µM trisodium citrate, 3 µM MgSO_4_, 2% glucose, 0.2% K-glutamate, 0.1% casein hydrolysate, 22 µg/mL ferric ammonium citrate, and 50 µg/mL tryptophan.

### Swarming and swimming cell expansion assays

Swarming motility was detected as described before (47). Cultures were grown aerobically in LB broth at 37°C to OD_600_ ~0.5. 1 mL of cultures were resuspended in 50 µL of 1× PBS buffer with 0.5% India link. 10 µL of the resuspended cells was inoculated on the center of standard (100 × 15 mm) LB agar plates (for complementation, 0.5% of xylose was used for induction). LB plates were made freshly before cell incubation. Each plate contained 25 mL of LB medium with different agar concentrations (0.5%–0.9%) and was dried for 10 min under a laminar flow hood. The plates with inoculated cells were dried for 15 min and incubated at 37˚C. The origin of the plate was demarked by the India ink, and the swarming radius was measured from the origin to the edge of swarming along the marked axis which was drawn on the back of the plate at the beginning. To monitor swimming motility, a single colony of each desired strain was stabbed into the center of an LB plate supplemented with 0.3% agar and then incubated at 37˚C for 12 h. Images were taken with a BioRad Chemi doc.

### Flagellin detection by SDS-PAGE gel

Cells were grown in liquid LB medium at 37°C to OD_600_ ~0.5. 1 mL of cultures were washed by 1× PBS buffer and resuspended in 50 µL of 1× PBS buffer and 50 µL of 2× Laemmli sample buffer. All the resuspended samples were heated at 98˚C for 30 min and then spun down at 15,000 *× g* for 1 min. 12 µL of supernatants were analyzed by SDS-PAGE. After electrophoresis, the gels were stained by Coomassie Blue dyes for 1 hour, and then destained (10% acetic acid, 50% methanol, and 40% H_2_O) overnight. Images of gels were taken using a GelDoc Gel imaging system (Bio-Rad, USA). Band intensity was analyzed by ImageJ software.

### Basal body and cell length detected by microscopy

Mutation of MeeF and MeeY was transformed into strain 3610 ∆*fliM amyE::P_flache-fliM-GFP_*. The detailed method for FliM-GFP visualization using super-resolution fluorescence microscopy is described in a previous paper (25). In brief, GFP-labeled fluorescent puncta were quantified in cells lacking the endogenous *fliM* gene (to reduce background fluorescence), and cell bodies were visualized by membrane staining with FM4-64. For super-resolution microscopy, the OMX 3D-SIM Super-Resolution system was used with a 1.4-numerical-aperture (NA) Olympus 100× oil objective. FM4-64 was observed using laser line 561 and emission filter 609 nm to 654 nm, and GFP (along with Alexa Fluor 488 nm) was observed using laser line 488 nm and emission filter 500 nm to 550 nm. Images were captured using a Photometrics Cascade II electron-multiplying charge-coupled-device camera, processed using SoftWorx imaging software, and analyzed using Imaris software.

### Extracellular complementation

Cells were grown aerobically in LB broth at 37°C to OD_600_ ~1. Supernatants were first collected by centrifuging the cultures at 1,200 *× g* for 10 minutes, and then the supernatants were filtered through 0.22 µm Foxx polyethersulfone (PES) membrane filters (EZFlow^®^). Equal volumes of supernatants and fresh 1.4% LB agar were mixed and solidified in the plates for 10 min under a laminar flow hood. 5 µL of resuspended cultures, which were grown in LB to OD_600_ ~0.5 (resuspended in 1× PBS with 0.5% India ink), was spotted on the center of each mixing LB agar. Plates were incubated at 37˚C overnight, and images were taken.

Chemical complementation: bacterial cells were grown to a mid-log phase aerobically in LB broth at 37°C to OD_600_ ~0.3. 1 mL of cultures were resuspended in 50 µL of 1× PBS buffer with 0.5% India ink. 5 μL of cultures was spotted onto LB and LB containing 50 µg of surfactin (dissolved in DMSO). Plates were incubated at 37˚C and images were captured.

## SUPPLEMENTAL MATERIAL

Supplemental figures and tables jb.00088-25-s0001.docx Figure S1 to S5; Tables S1 to S3.

ASM does not own the copyrights to Supplemental Material that may be linked to, or accessed through, an article. The authors have granted ASM a non-exclusive, world-wide license to publish the Supplemental Material files. Please contact the corresponding author directly for reuse.
