# Supplementary material for: The TerC family metal chaperone MeeY enables surfactin export in Bacillus subtilis
Source: J Bacteriol. 2025 Apr 16;207(5):e00088-25. doi: 10.1128/jb.00088-25 (PMC12096827; doi:10.1128/jb.00088-25)
Supplement: Supplemental figures and tables — Figure S1 to S5; Tables S1 to S3. [file jb.00088-25-s0001.docx]

**The TerC family metal chaperone MeeY enables**

**surfactin export in *Bacillus subtilis***

Bixi He^1^, Ankita J. Sachla^1^, Sadie B. Ruesewald^2^, Daniel B. Kearns^2^, and John D. Helmann^1^*

^1^Department of Microbiology, Cornell University, Ithaca, NY, USA, 14853-8101

^2^Department of Biology, Indiana University, Bloomington, IN 47408, USA.

**SUPPLEMENTAL MATERIAL**

Figure S1. Swarm plate assays of strains lacking *meeY*, *meeF*, or both compared to a *hag* mutant strain

Figure S2. Ectopic expression of *meeY* can support swarming

Figure S3. Swimming motility of *meeF*, *meeY*, and FY mutants

Figure S4. Flagellin levels in *meeF*, *meeY*, and FY mutants

Figure S5. Extracellular swarm complementation of the FY mutant with supernatants

Table S1. Basal body and cell length of WT, *meeF, meeY*, and FY mutants

Table S2. *Bacillus subtilis* stains used in this study

Table S3. Primers used in this study

**Supplementary Figures**

**
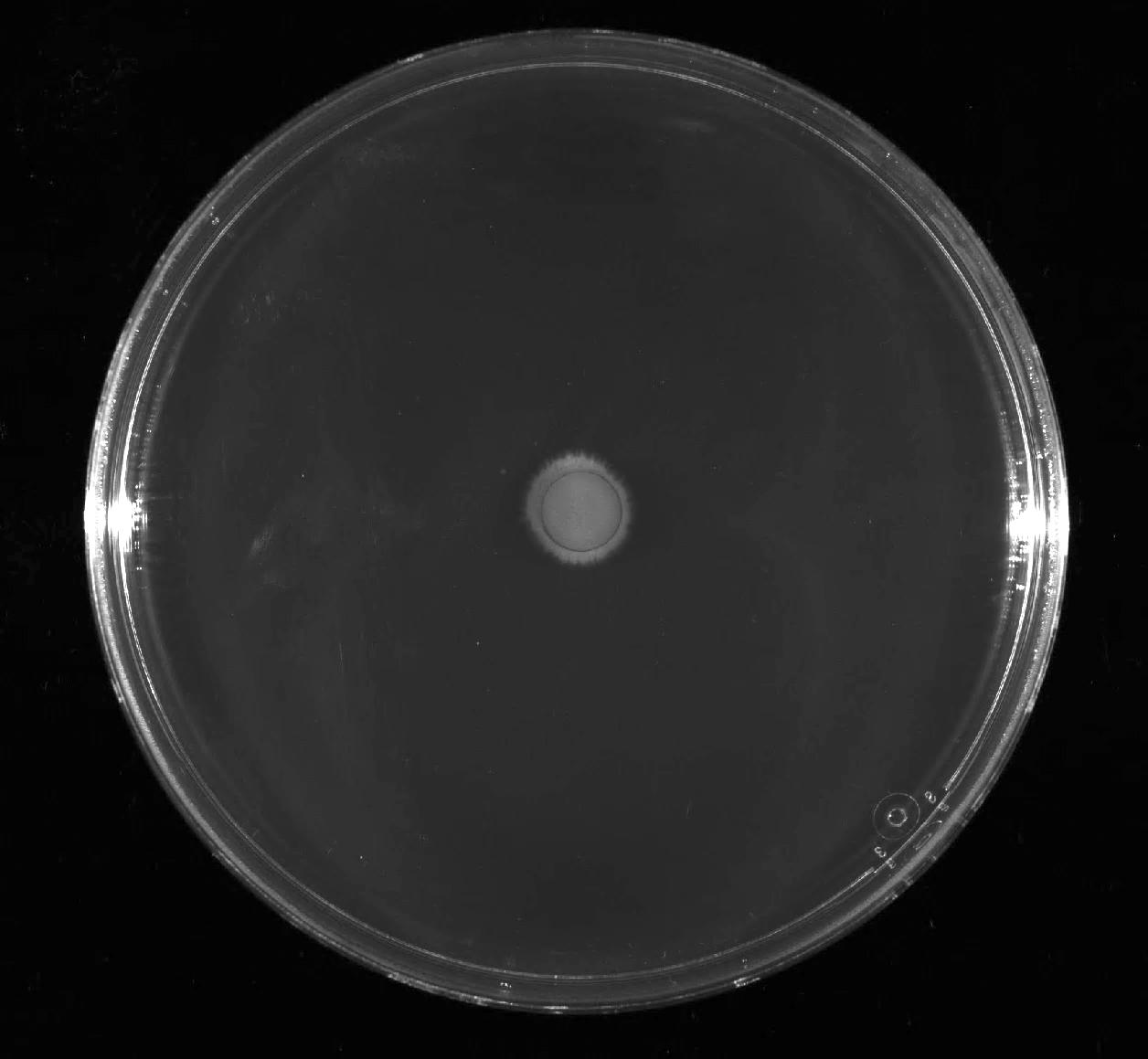

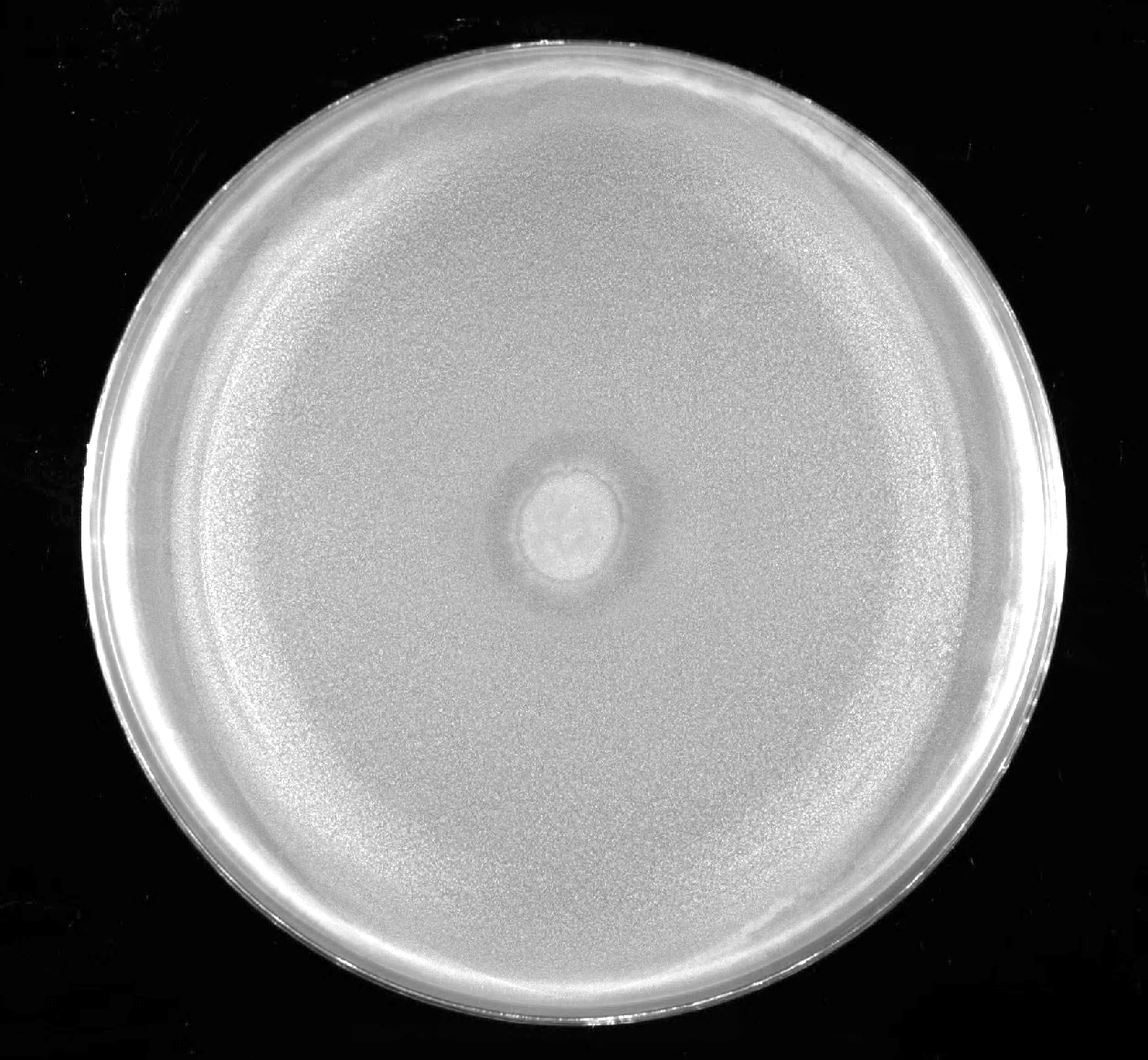

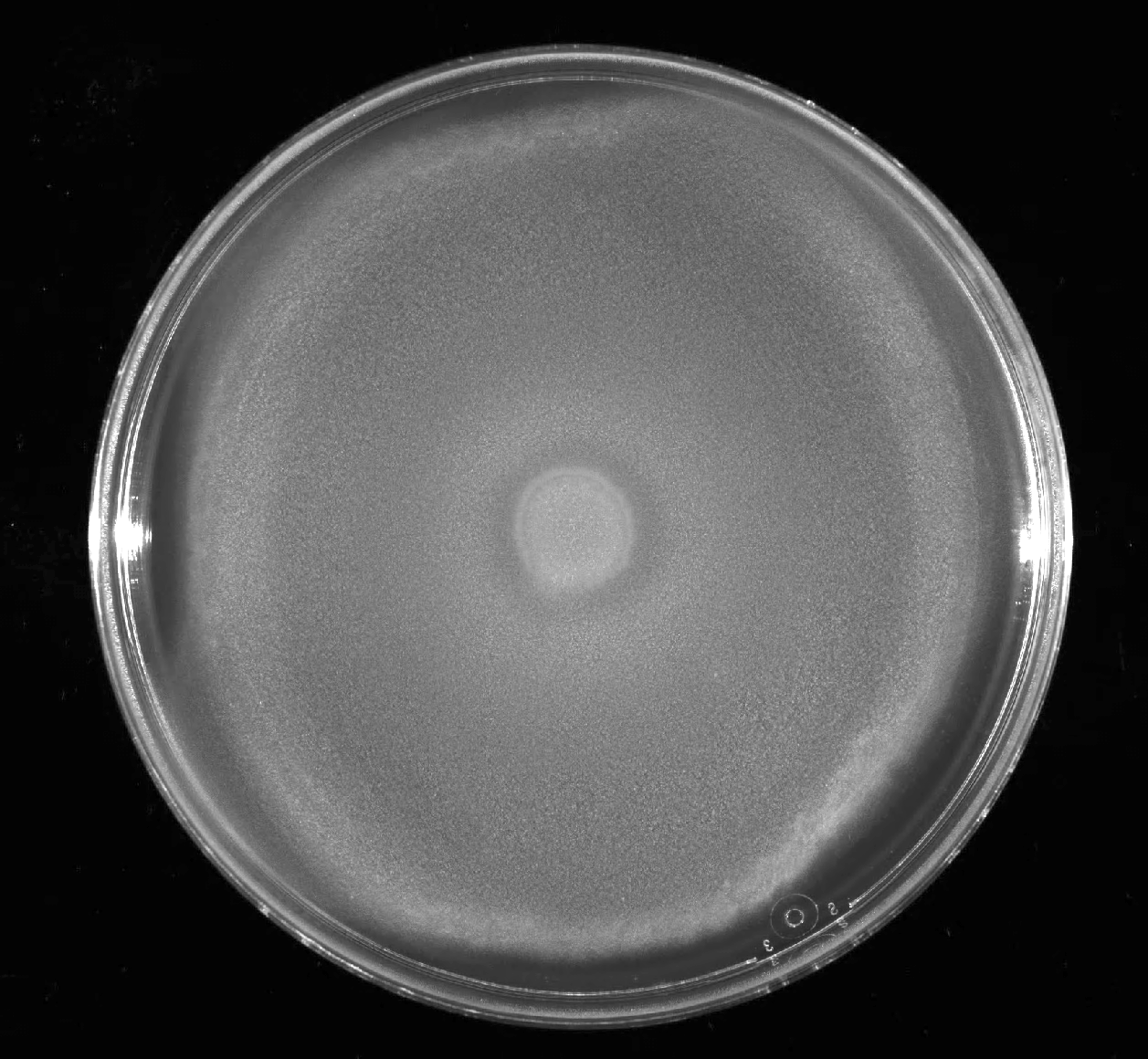

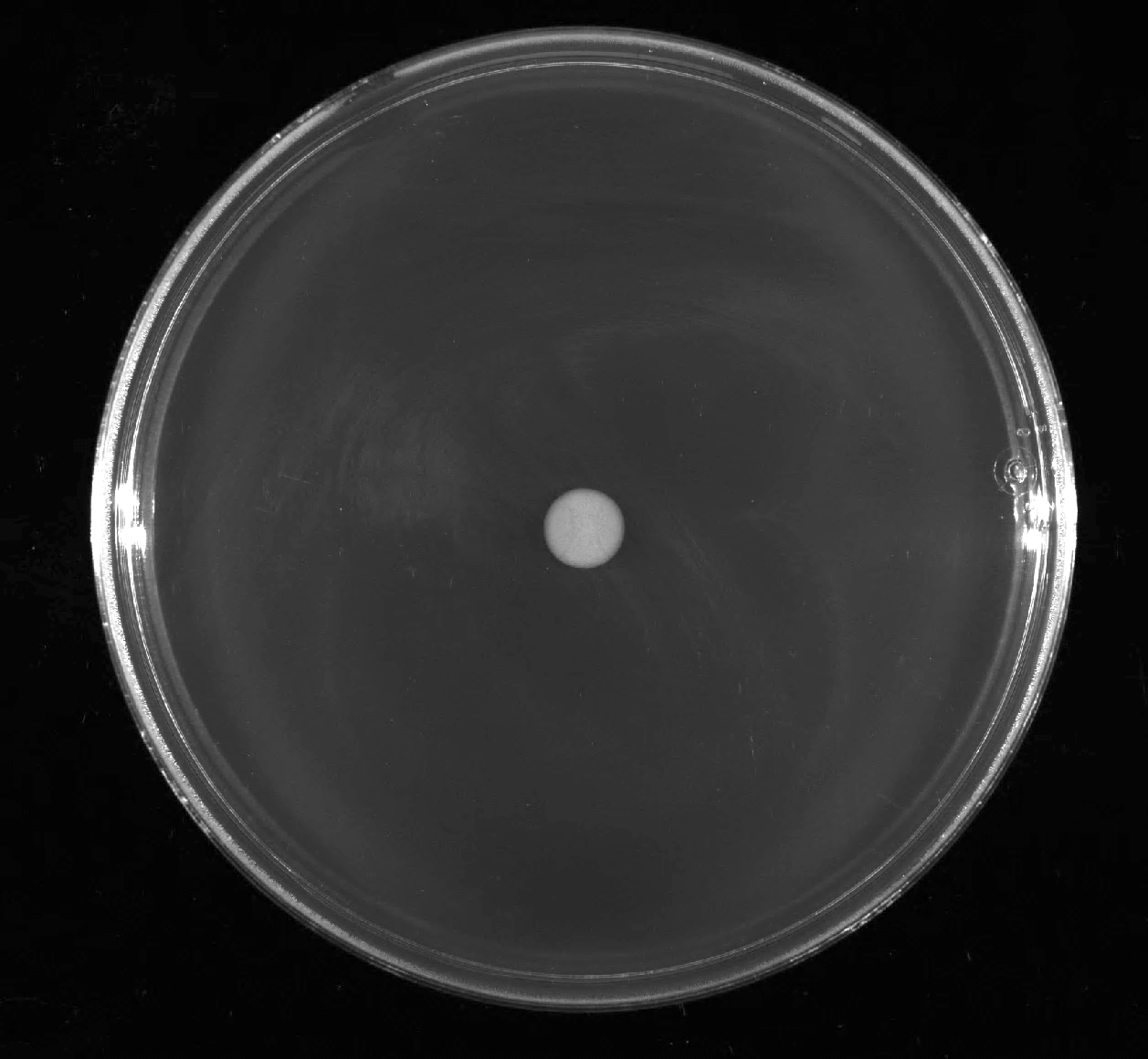

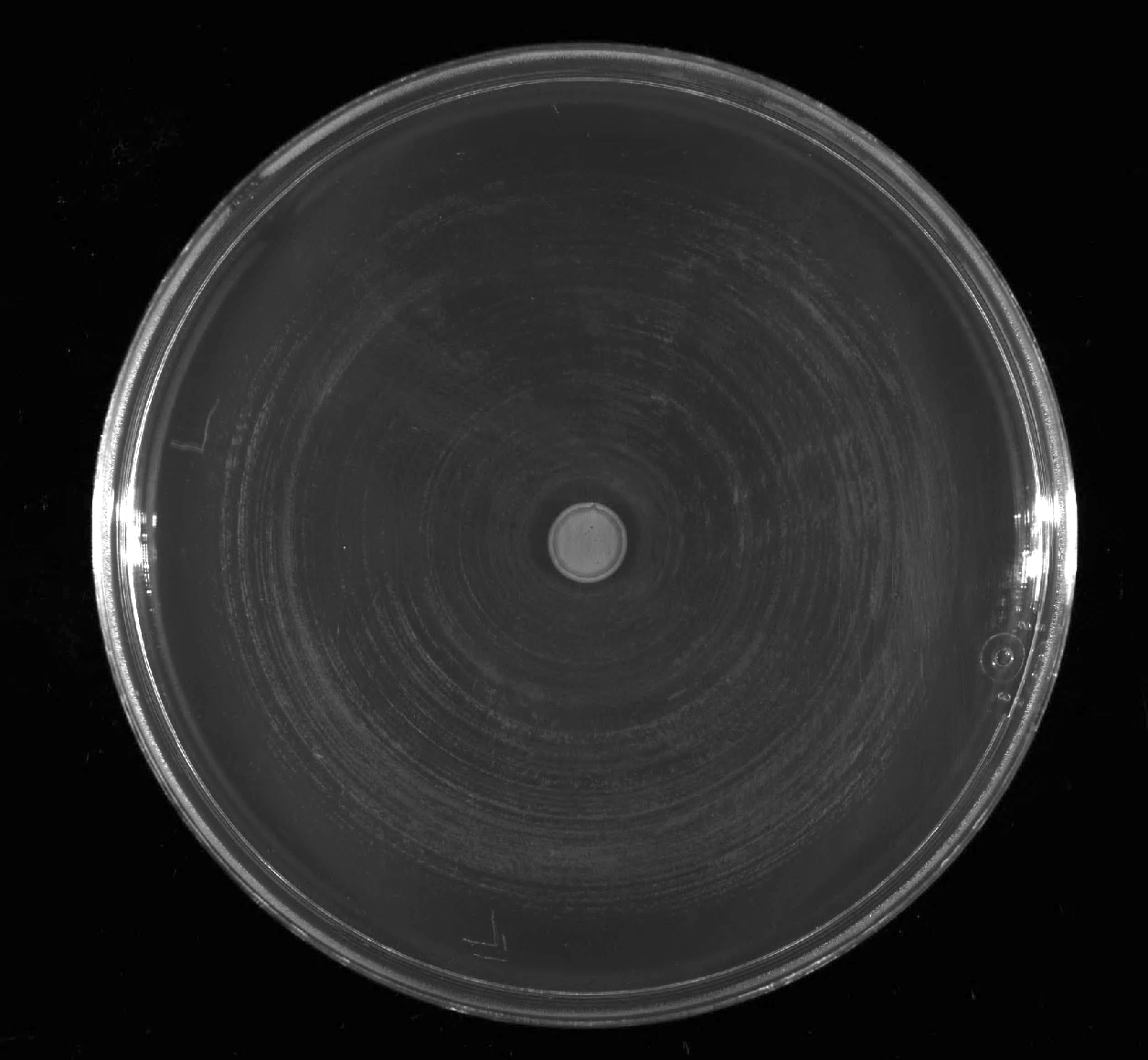
**

***hag::erm***

**3610**

***meeF::kan***

***meeY::kan***

***FY***

**Figure S1. Swarm plate assays of strains lacking *meeY*, *meeF*, or both compared to a *hag* mutant strain.** Swarm plates were imaged after 8 hrs.


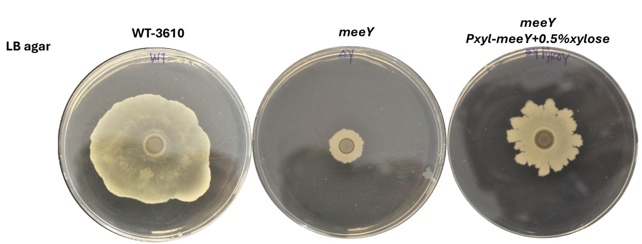


**Figure S2. Ectopic expression of *meeY* can support swarming.** The indicated stains were spotted on swarm plates containing 0.5% xylose (to induce *meeY* expression in the complemented strain) and then plates were visualized after 8 hrs.


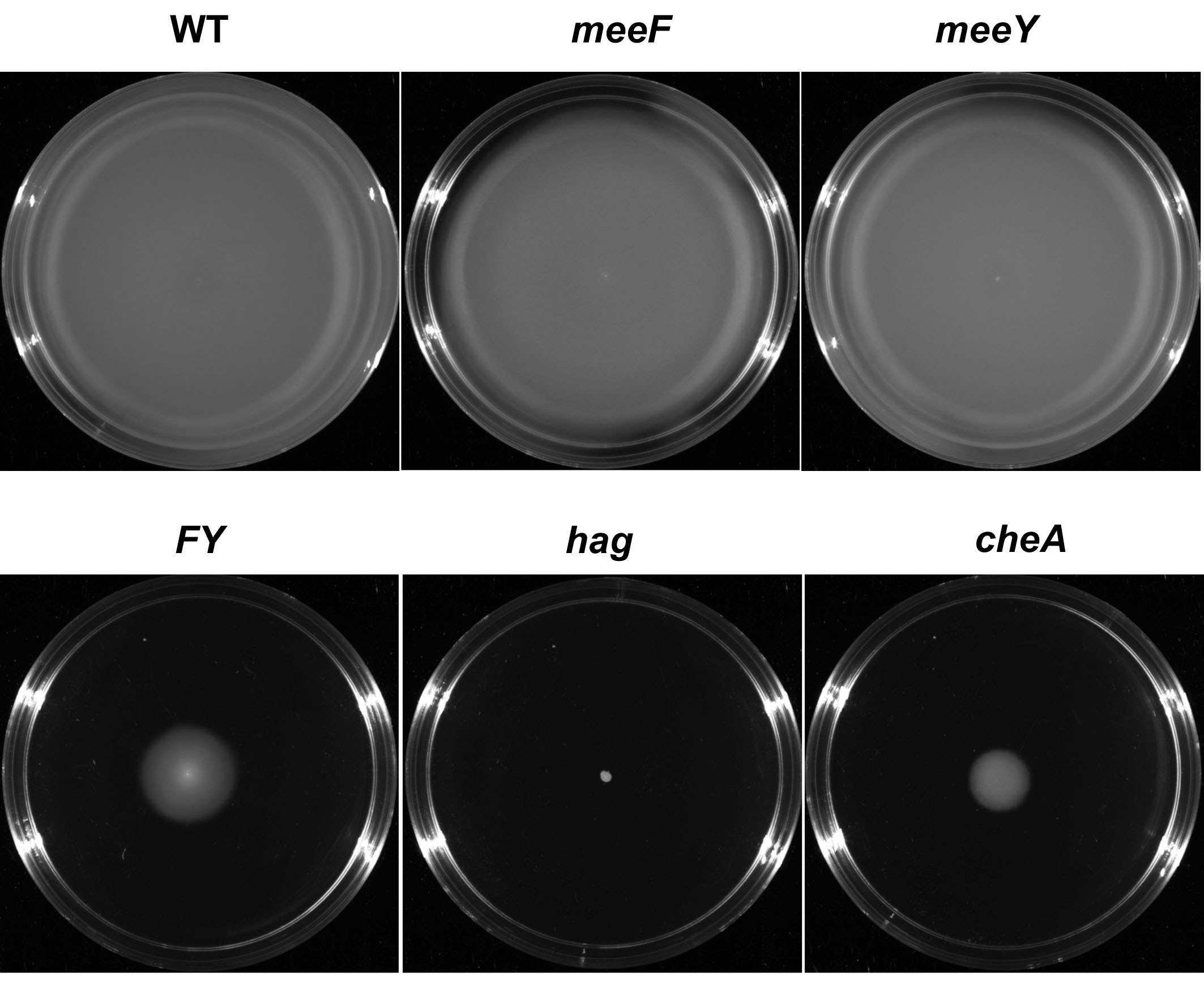


**Figure S3. Swimming motility of *meeF*, *meeY*, and FY mutants.** Strains were visualized after 12 hrs at 37 ˚C.


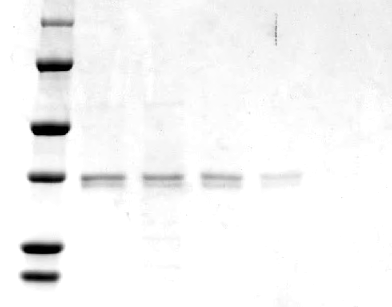


**37**

**50**

**25**

**20**

**100**

**75**

**M**

**3610**

***meeF::kan***

***meeY::erm***

***FY***

***hag::erm***

**Figure S4. Flagellin levels in *meeF*, *meeY*, and FY mutants.** Cells were heated in the presence of SDS sample buffer at 98 ˚C. In the absence of lysozyme, there is minimal cell lysis and the protein bands near ~37 kDa by subsequent Coomassie-stained SDS-PAGE analysis are flagellin (Hag) and, as expected, are absent in the *hag* mutant. Band intensity change was calculated as “change = (sample - control) / control * 100%”. The Image is representative of four independent experiments and band intensities were measured relative to WT. In this example, the decrease in flagellin abundance was ~18% for *meeF*, ~41% for *meeY*, and ~73% for the FY double mutant.


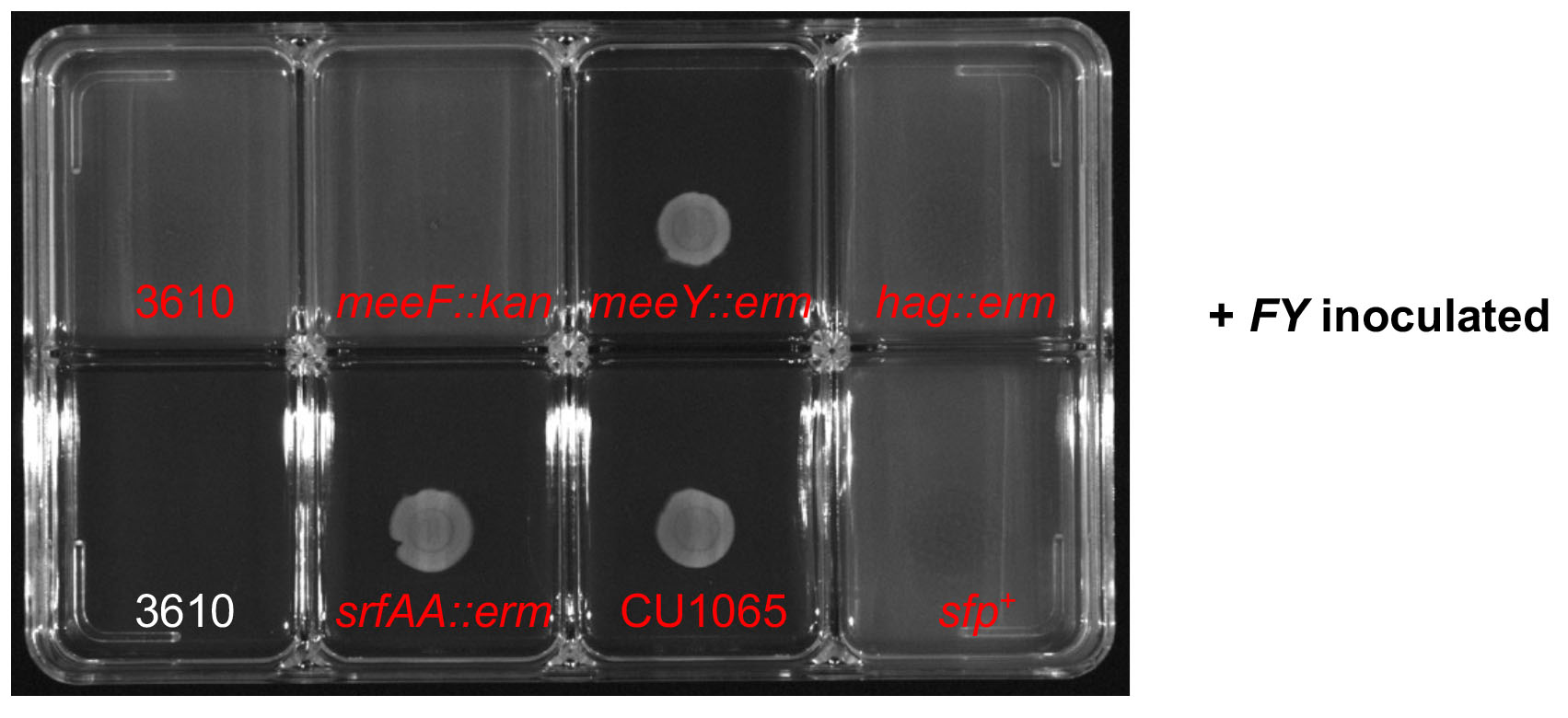


**Figure S5. Extracellular swarm complementation of the FY mutant with supernatants.** Supernatants were collected from different cultures (grown in LB to OD600~1), filter-sterilized, and tested for their ability to restore swarming to spot-inoculated *FY* mutant cells (5 µl). The source of the supernatant used is indicated on the plate in red. The supernatants were from 3610 (upper left) or derivative strains, except for CU1065 and a CU1065 *sfp*^+^ derivative (bottom row). The lower left panel is a sterility control with 3610 supernatant only (white font) with LB agar and no cells added.

**Table S1**. **Basal body and cell length of WT, *meeF, meeY*, and FY mutants.** There are 40 individual cells counted for each strain and data are presented as mean ± standard deviation.

| **Strain** | **Basal Body (#)** | **Cell Length (µm)** |
| --- | --- | --- |
| **WT** | 20.55 ± 4.16 | 3.70 ± 0.62 |
| ***meeF*** | 17.95 ± 3.16 | 4.21 ± 0.83 |
| ***meeY*** | 19.95 ± 2.74 | 4.42 ± 0.71 |
| ***FY*** | 17.53 ± 5.80 | 4.73 ± 0.96 |

**Table S2. *Bacillus subtilis* stains used in this study**

| **Strain** | **Genotype** | **Construction** | **Reference** |
| --- | --- | --- | --- |
| CU1065 (HB27501) | *trpC2 attSPβ (WT)* | Lab strain | (1) |
| HB27502 | *CU1065 ΔmeeF* | Lab strain | Lab stock |
| HB5800 (HB31518) | *CU1065 sfp^+^* | *sfp*^+^ introduced by congression | (2) |
| HB27503 | *trpC2* Δ*meeY* | Lab strain | Lab stock |
| HB27504 | *trpC2* Δ*meeF* Δ*meeY* | Lab strain | Lab stock |
| HB27928 | *NCIB* 3610 Δ*fliM amyE::P_flache-_fliM-GFP* |  | (3) |
| HB27955 | *NCIB* 3610 Δ*fliM amyE::P_flache-_fliM-GFP meeF:kan* | BGSC🡪HB27928 | This work |
| HB27957 | *NCIB* 3610 Δ*fliM amyE::P_flache-_fliM-GFP meeY::erm* | BGSC🡪HB27928 | This work |
| HB27959 | *NCIB* 3610 Δ*fliM amyE::P_flache-_fliM-GFP meeF:kan meeY::erm* | BGSC🡪HB27928 | This work |
| HB27754 | NCIB 3610 |  | Lab stock |
| HB27755 | *NCIB* 3610 *meeF::kan* | SPP1 transduction | This work |
| HB27761 | *NCIB* 3610 *meeY::erm* | SPP1 transduction | This work |
| HB27768 | *NCIB* 3610 *meeF::kan meeY::erm* | SPP1 transduction | This work |
| HB27996 | *NCIB 3610 hag::erm* | SPP1 transduction | This work |
| HB31514 | *NCIB 3610 srfAA::erm* | SPP1 transduction | This work |
| HBYL1823 | NCIB 3610 *meeY*::kan *lacA*::P_xyl_-*meeY* | pAX01-*meeY*🡪*meeY*::*kan* | This work |
| DK1485 | *NCIB* 3610 *srfAC::Tn10 spec, ΔespH* |  | (4) |
| DK2203 | *NCIB 3610 srfAC::Tn10 spec, epsH::tet, ΔcheA* |  | (4) |
| DB2245 | *NCIB 3610 srfAC::Tn10 spec, ΔespH, hag::kan* | DK1485 🡪 *hag::kan* | This work |
| DB2243 | *NCIB 3610 srfAC::Tn10 spec, ΔespH, meeY::erm* | DK1485 🡪 *meeY::erm* | This work |
| DB2244 | *NCIB 3610 srfAC::Tn10 spec, ΔespH, meeF::kan*  *amyE::P_hyspank-motAB_ meeF::kan meeY::erm* | DK1485 🡪 *meeF::kan* | This work |
| DB2322 | *NCIB 3610 srfAC::Tn10 spec, ΔespH, meeF::kan, meeY::erm* | DB2244 🡪 *meeY::erm* | This work |

**Table S3. Primers used in this study.**

| **Primer name** | **Sequence (5’ to 3’)** | **Purpose** |
| --- | --- | --- |
| meeFcheckF | GTGTCATCCATACGGGTGACAA | Deletion check |
| meeFcheckR | GATTTGAGTGCTTCGCAATCAGCT |  |
| meeYcheckF | GGAGGAAGGCCGTTTCTTGA | Deletion check |
| meeYcheckR | CGGATGAAACCGTCTTTGCG |  |
| hagcheckF | ACAAAATCAGAGACAATCCGATATTAATGATGT | Deletion check |
| hagcheckR | CGACTAAGTCGAGATAAGATTACACATATTTTTGG |  |
| srfAAcheckF | attggaagcactgctttttaagtgtagt | Deletion check |
| srfAAcheckR | gatcaagcatcgcatgatacagcat |  |
| fliMcheckF | CTGATACGAATGCTGATCAGATTGAGG | Deletion check |
| fliMcheckR | GAAATATTACCGATTTCACCGATCGCAT |  |
| SpeI-ykoY-F | atatACTAGTaatcatatttcttgaaatcc | Complement |
| BamHI-ykoY-R | cgcgGGATCCttacgcccgttcacgggtgctgtttttttg | Complement |

**Supplemental References:**

1. Zahler SA, Korman RZ, Rosenthal R, Hemphill HE. 1977. *Bacillus subtilis* bacteriophage SPbeta: localization of the prophage attachment site, and specialized transduction. J Bacteriol 129:556-8.

2. Ollinger J, Song K-B, Antelmann H, Hecker M, Helmann John D. 2006. Role of the Fur Regulon in Iron Transport in *Bacillus subtilis*. Journal of Bacteriology 188:3664-3673.

3. Guttenplan SB, Shaw S, Kearns DB. 2013. The cell biology of peritrichous flagella in *Bacillus subtilis*. Mol Microbiol 87:211-29.

4. Hall Ashley N, Subramanian S, Oshiro Reid T, Canzoneri Alexandra K, Kearns Daniel B. 2017. SwrD (YlzI) Promotes Swarming in *Bacillus subtilis* by Increasing Power to Flagellar Motors. Journal of Bacteriology 200:10.1128/jb.00529-17.
